# Supplementary material for: Characterization of Slurry-Cast Layer Compounds for 3D Printing of High Strength Casting Cores
Source: Materials (Basel). 2021 Oct 16;14(20):6149. doi: 10.3390/ma14206149 (PMC8539653; doi:10.3390/ma14206149)
Supplement: Supplementary file 1 [file materials-14-06149-s001.zip › materials-1393455-supplementary.pdf]

materials

Supplementary Materials

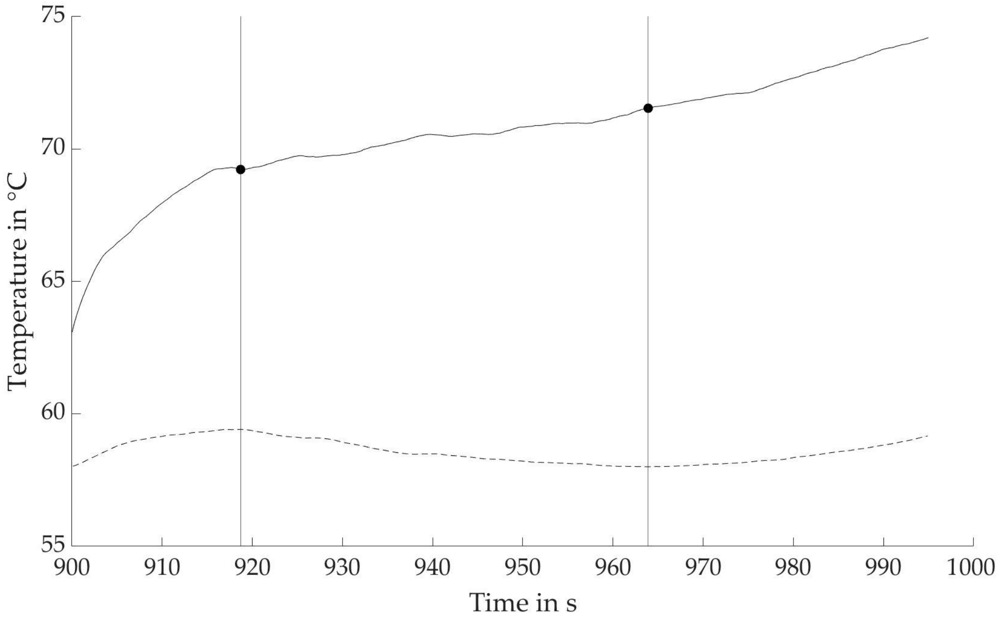

Figure S1. Determination of Drying Points for PAV = 3.8 V.

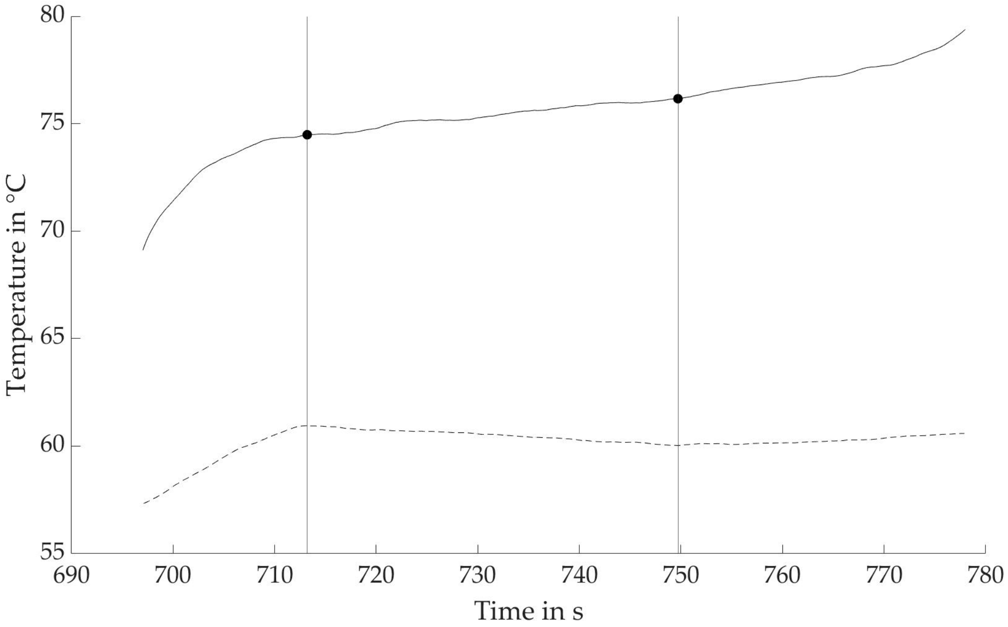

Figure S2. Determination of Drying Points for PAV = 4.2V.
